# Supplementary material for: The child Musicality Index: A child-friendly version of the Goldsmiths Musical Sophistication Index
Source: PLoS One. 2026 Jan 23;21(1):e0339261. doi: 10.1371/journal.pone.0339261 (PMC12829873; doi:10.1371/journal.pone.0339261)
Supplement: S3 File — (DOCX) [file pone.0339261.s003.docx]

**S3 Appendix**

**Response-validity analysis from study 1a.**

Study 1a identified 8-items to create a new scale. The current analysis was carried out as part of this study, and aimed to investigate the internal validity of children’s responses to these items by following an approach documented by Conijn et al. [1]. Extreme response bias, or frequent use of the extreme ends of a Likert scale, has been reported with young children (5-6 years [2]) who are more likely to provide overestimates of their self-perceived ability than older children [3,4]. Studies have also detected a nondifferentiation bias in this young age group (<7 years), meaning they are more likely to provide random responses or the same response for every item [5]. The current investigation was carried out to determine whether children exhibited these biased response patterns answering the 8 items which would make up the new scale.

A ‘validity-indexing’ approach [1] was applied to determine whether age had an effect on how children in the current sample responded to the selected items.

This study therefore examined whether children’s age affected the internal validity of the proposed scale. On the basis of previous findings [1,2] we expected that 6-year-olds completing the items would exhibit higher levels random/inconsistent and extreme responding.

**Method**

Refer to the method section of study 1a for details.

**Analysis**

The current study aimed to examine the effects of age on the validity of children’s responses. Data were taken from study 1b meaning that children had seen a selection of 48 possible items, however the current analyses only considered responses to the 8 items which had been selected to produce the new, short scale.

The validity-indexing approach [1] assesses response validity by calculating estimates of random/inconsistent responding in a response pattern. Estimates are based on the number Guttman errors detected (see [6]). A Guttman error occurs when a participant selects a response to an item which has not been selected frequently across the rest of the sample. *G^p^* and *G^p^_n_* indices are calculated for each participant based on the number of Guttman errors. *G^p^* scores can range between 0 and the total test score of a given participant. *G^p^_n_* scores are normed and range from 0 to 1, meaning they account for the possible maximum value of *G^p^* given the participants total score, and are thus better able to compare participants with different ability levels. In both cases, high scores represent higher inconsistency. To adhere to assumptions of the *G^p^* index as an indicator of validity, the group factor data must be related by a single strong dimension. We computed eigenvalues using the *fa.parallel* function from the psych package in R [7] to assess the unidimensionality of each identified factor. Following this we calculated *G^p^* indices for each child using the *Gpoly* function from the Perfit package in R [8].

*G^p^* statistics for the full 8-item scale and the two 3-item subscales were calculated for each child. We took the mean of three *G^p^* scores gathered from the two subscales and general factor as the final measure of level of random/inconsistent responding for each child. The *G^p^_n_* statistic could not be computed for the subscales due to the small number of items and was not further investigated.

We additionally calculated a cutoff threshold based on the distribution of bootstrapped *G^p^* scores, past which point the children’s responses could be classified as invalid. The bootstrap procedure used 10000 replications to estimate the mean and SE of resampled data. We set the cut-off at a level of 2 SE above the mean, following recommendations from Conijn et al. [1]. This was used to create a new binary variable ‘classification rate’ which indicated whether children’s *G^p^* score was above this threshold and should therefore be classified as considered ‘severely inconsistent’.

An extreme response bias index [9] was also extracted as an estimate of response validity, as recommended by Conijn et al. [1]. This index was calculated by summing the number of responses at the extreme ends of the Likert scale for each participant.

Once indicators of response validity had been estimated, regression analyses were carried out to evaluate the effect of age group and task environment on each indicator. The *lm* and *glm* functions in base R were used to build linear and logistic regression models, respectively.

**Results**

Estimates of random/inconsistent and extreme responding were calculated for the 283 children who provided 3 or more responses to the 8 selected items.

Assumption checks were carried out before calculating *G^p^* scores to estimate random/inconsistent responding. We found evidence for unidimensionality of the general factor based on 41% of variance in item scores being explained by the first component and a ratio of <1/3 (=.17) between the first and second eigenvalue [10]. Each of the subscales contained only three items which meant computing more than one eigenvalue was not possible. We thus had to assume unidimensionality for both subscales without further checks.

We investigated whether response-validity, as indicated by random/inconsistent responding, classification rate, and extreme responding, could be explained by age. To account for a non-linear relationship between age and all indicators of response validity, we included a squared term of the age variable in each of the model formulae. Formulae for each model are included in the R markdown file “suppl_study1a_responsevalidity.Rmd” which can be accessed via the OSF repository [11].

Regression models revealed a significant negative effect of the linear age term on *G^p^* scores (*b*=-6.9, *t*=-3.5, R^2^=.063*, p*<.001) and extreme responses (*b*=-1.6, *t*=-2.43, R^2^=.045, *p*<.05). Visual inspection indicated that 6-year-olds provided the highest level of random/inconsistent response patterns according to these two indices, followed by 7-year-olds (see Fig 2 and 3). Unexpectedly, the oldest age group (13 years) also displayed high levels of random/inconsistent responding compared with 8- to 12-year-olds. A logistic regression model revealed a significant negative effect of age on responses classified as severely inconsistent (*b*=-4.78, *z*=2.24, *p*<.001; see Fig 3), following a similar pattern with 6 and 7-year-olds exhibiting the highest classification rates.

**
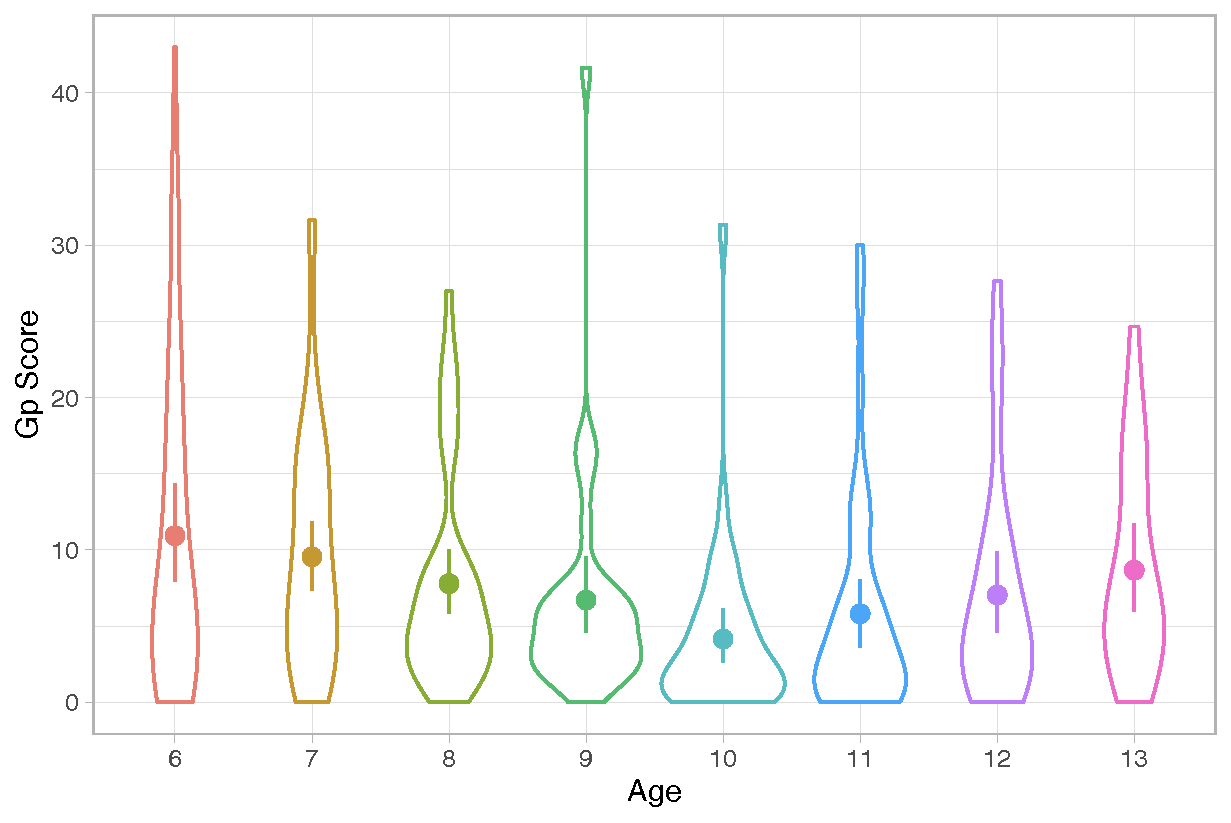
**

**Fig 2. Level of random/inconsistent responding (*G^p^* score) by age group.**

Violin shapes represent the data distribution, dots represent the mean, bars represent SE.

**Fig 3.** **Number of extreme responses by age group.**

**
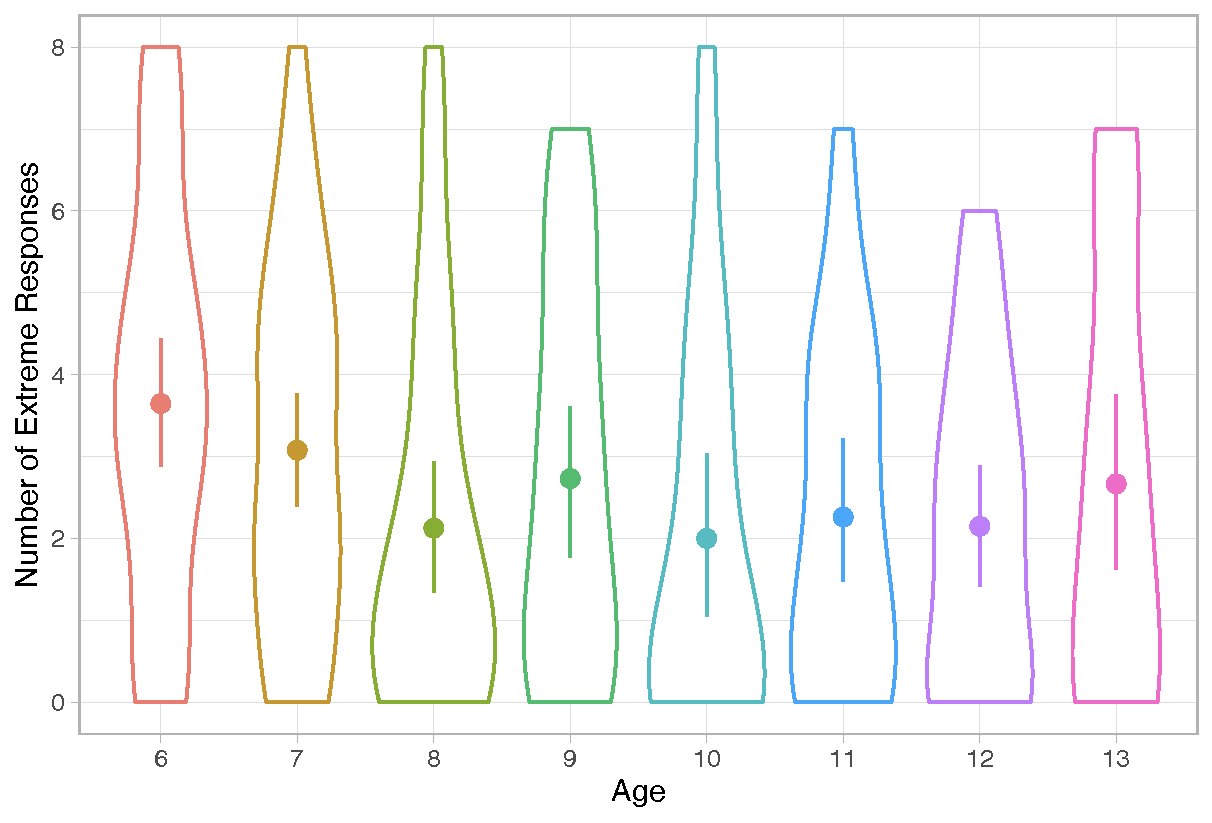
**

Violin shapes represent the data distribution, dots represent the mean, bars represent SE.

**
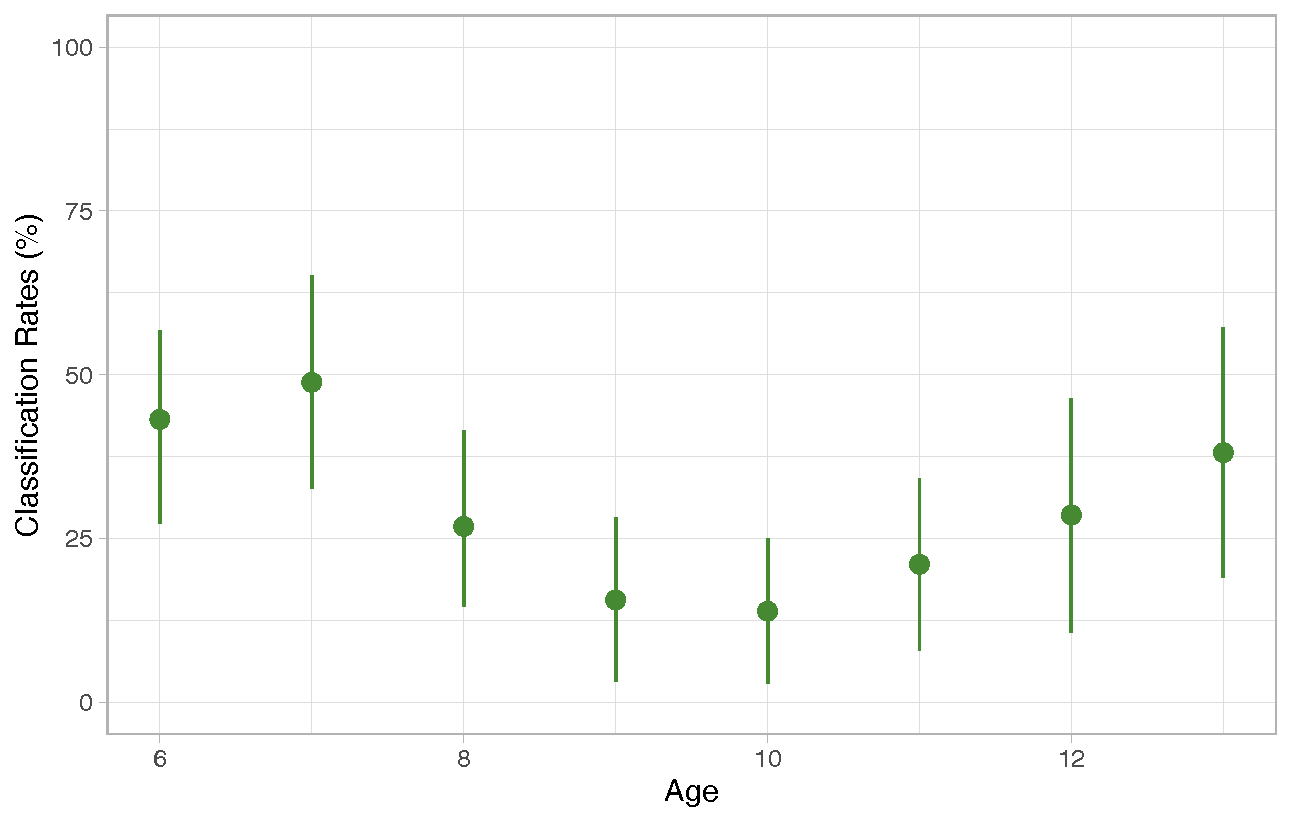
**

**Fig 4. The proportion of response patterns classified as random/inconsistent (classification rates) by age group.**

Dots represent the mean, bars represent SE.

Violin shapes represent the data distribution, dots represent the mean, bars represent SE.

In sum, these findings suggest that responses from youngest participants (6 & 7 years) and oldest participants (13 years) show higher levels of random and extreme responding compared to the data from 8- to 12-year-olds.

**References**

1. Conijn JM, Smits N, Hartman EE. Determining at What Age Children Provide Sound Self-Reports: An Illustration of the Validity-Index Approach. Assessment. 2020 Oct 1;27(7):1604–18.

2. Chambers CT, Johnston C. Developmental Differences in Children’s Use of Rating Scales. Journal of Pediatric Psychology. 2002 Jan 1;27(1):27–36.

3. Eccles J, Wigfield A, Harold RD, Blumenfeld P. Age and Gender Differences in Children’s Self- and Task Perceptions during Elementary School. Child Development. 1993;64(3):830–47.

4. Jacobs JE, Lanza S, Osgood DW, Eccles JS, Wigfield A. Changes in Children’s Self-Competence and Values: Gender and Domain Differences across Grades One through Twelve. Child Development. 2002;73(2):509–27.

5. Shelton KK, Frick PJ. Assessment of parenting practices in families of elementary school-age children. Journal of Clinical Child Psychology. 1996 Sept;25(3):317.

6. Meijer RR. The Number of Guttman Errors as a Simple and Powerful Person-Fit Statistic. Applied Psychological Measurement. 1994 Dec 1;18(4):311–4.

7. Revelle W. psych: Procedures for Psychological, Psychometric, and Personality Research. [Internet]. Northwestern University, Evanston, Illinois.; 2023. Available from: https://CRAN.R-project.org/package=psych

8. Tendeiro J, Meijer R, Neissen S. PerFit: An R Package for Person-Fit Analysis in IRT. Journal of Statistical Software. 2016;74(5):1–27.

9. van Herk H, Poortinga YH, Verhallen TMM. Response Styles in Rating Scales: Evidence of Method Bias in Data From Six EU Countries. Journal of Cross-Cultural Psychology. 2004 May 1;35(3):346–60.

10. Slocum-Gori SL, Zumbo BD. Assessing the Unidimensionality of Psychological Scales: Using Multiple Criteria from Factor Analysis. Soc Indic Res. 2011 July 1;102(3):443–61.

11. MacGregor C, Mednicoff S, Vollweiler DJ, Hannon E and Müllensiefen D. The child Musicality Index: a child-friendly version of the Goldsmiths Musical Sophistication Index [Internet]. OSF; Available from: https://osf.io/jgrk6/overview?view_only=bf376f77b1d945d8aa32f1bbed7df4f2
