# Supplementary material for: The child Musicality Index: A child-friendly version of the Goldsmiths Musical Sophistication Index
Source: PLoS One. 2026 Jan 23;21(1):e0339261. doi: 10.1371/journal.pone.0339261 (PMC12829873; doi:10.1371/journal.pone.0339261)
Supplement: S4 File — (DOCX) [file pone.0339261.s004.docx]

**S4 Appendix**

**Response-validity analysis from study 2.**

Study 2 analysed responses from a sample who completed the new 8-item scale (N=56). The current analysis was carried out as part of this study, and aimed to investigate whether children’s responses to these items exhibited random or extreme response patterns. Refer to Appendix S3 for a full outline of the approach.

On the basis of the findings of study 1a, and findings from previous literature [1,2] we expected that 6-year-olds completing the items would exhibit higher levels random/inconsistent and extreme responding.

**Method**

Refer to the method section of study 2 for details.

**Analysis**

The current study aimed to examine the effects of age on the validity of children’s responses. Data were taken from study 2 meaning that children had seen the 8-item cMI. Classification rates to be used as a cut-off for random/inconsistent responding could not be meaningfully estimated as they were in study 1a, due to the smaller sample in the current study.

**Results**

Estimates of random/inconsistent and extreme responding were calculated for the 56 children who took part in study 2.

Visual inspection indicated that 6- and 7-year-olds demonstrated similar levels of random and extreme responding as children in older age groups (see Fig 1 and 2).

**
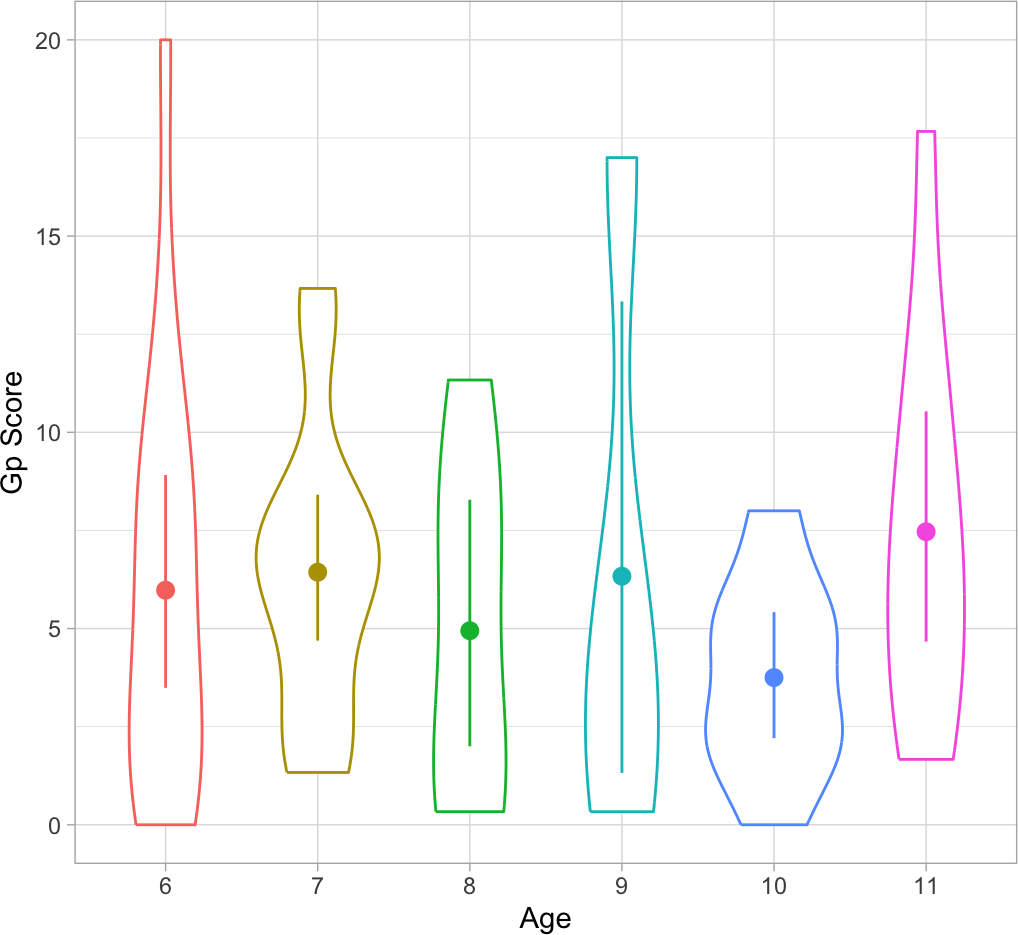
**

**Fig 1. Level of random/inconsistent responding (*G^p^* score) by age group.**

Violin shapes represent the data distribution, dots represent the mean, bars represent SE.

**Fig 2.** **Number of extreme responses by age group.**

**
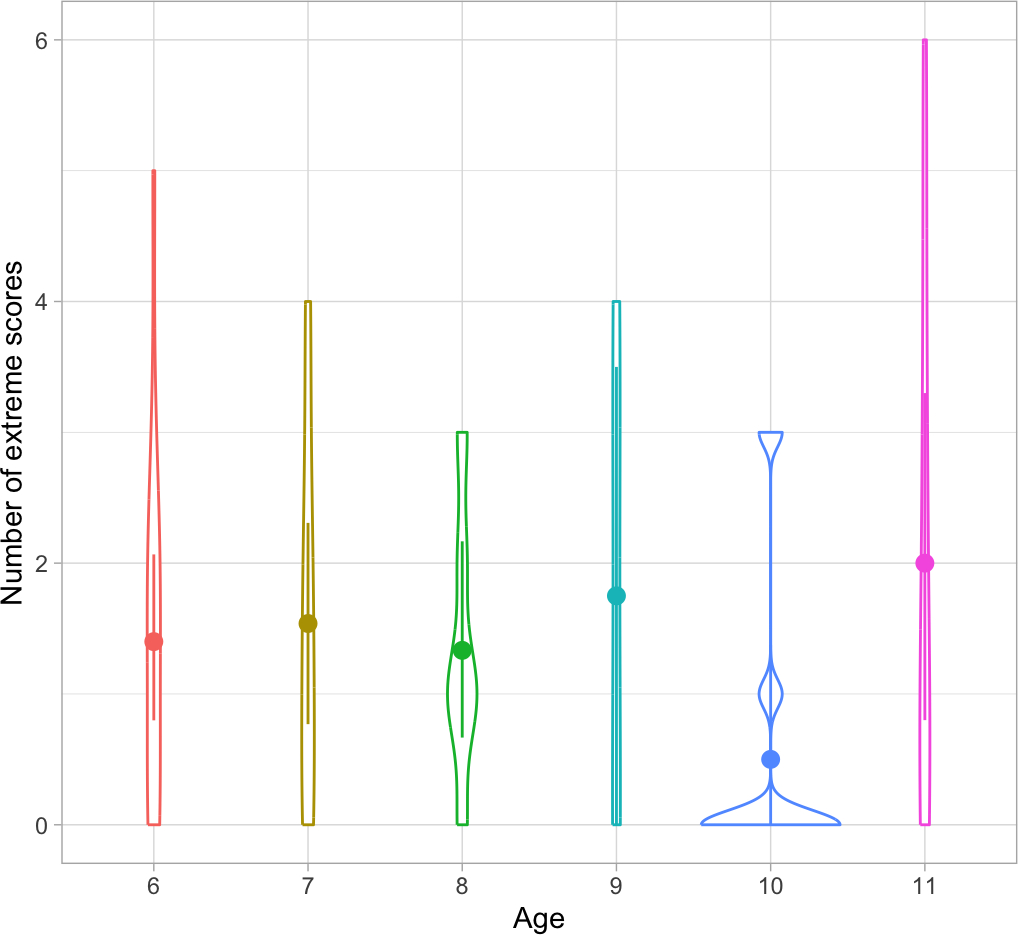
**

Violin shapes represent the data distribution, dots represent the mean, bars represent SE.

In sum, these findings suggest that youngest participants (6 & 7 years) demonstrated similar levels of random and extreme responding as children in older age groups.

**References**

1. Conijn JM, Smits N, Hartman EE. Determining at What Age Children Provide Sound Self-Reports: An Illustration of the Validity-Index Approach. Assessment. 2020 Oct 1;27(7):1604–18.

2. Chambers CT, Johnston C. Developmental Differences in Children’s Use of Rating Scales. Journal of Pediatric Psychology. 2002 Jan 1;27(1):27–36.
